# Identification of valid reference genes during the differentiation of human myoblasts

**DOI:** 10.1186/1471-2199-10-66

**Published:** 2009-07-02

**Authors:** Jens Stern-Straeter, Gabriel A Bonaterra, Karl Hörmann, Ralf Kinscherf, Ulrich R Goessler

**Affiliations:** 1 Department of Otolaryngology, Head and Neck Surgery, University Hospital Mannheim, University of Heidelberg, 68167 Mannheim, Germany; 2 Centre for Biomedicine and Medical Technology Mannheim (CBTM), Ludolf-Krehl-Str. 13-17, Tridomus, Building C, 68167 Mannheim, Germany

## Abstract

**Background:**

Analysis of RNA expression using real-time PCR (qRT-PCR) traditionally includes reference genes (RG) as an internal control. This practice is being questioned as it becomes increasingly clear that RG may vary considerably under certain experimental conditions. Thus, the validity of a particular RG must be determined for each experimental setting. We used qRT-PCR to measure the levels of six RG, which have been reported in the literature to be invariant. The RG were analyzed in human myoblast cultures under differentiation conditions. We examined the expression by qRT-PCR of mRNA encoding Beta-actin (ACTB), Beta-2-microglobulin (B2M), glyceraldehyde-3-phosphate dehydrogenase (GAPDH), peptidylprolyl isomerase A (PPIA), TATA box binding protein (TBP) and ribosomal protein, large, P0 (RPLPO). The mRNA expression of the following genes of interest (GOI) were analyzed: skeletal muscle alpha 1 actin (ACTA1), myogenin/myogenic factor 4 (MYOG), embryonic skeletal muscle myosin heavy chain 3 (MYH3) and the activity of creatine phosphokinase (CK). The geNorm, NormFinder and BestKeeper software programs were used to ascertain the most suitable RG to normalize the RNA input.

**Results:**

Using the geNorm program, RPLPO and TBP were found to be the most stable genes, additionally a suitable normalization factor (NF) was calculated. The NormFinder software showed that RPLPO was the most stable, whereas TBP ranked second. BestKeeper program also revealed that RPLPO and TBP as stable genes, but PPIA was the most stable reference gene, whereas GAPDH and ACTB were the worst ranked.

**Conclusion:**

RNA expression analyses including three independent softwares revealed that RPLPO, TBP as reference genes or NF calculated by geNorm software, are suitable to normalize the mRNA expression in myoblast after culture under differentiation conditions. Significant correlations can be identified between the differentiations markers ACTA1, MYOG, MYH3 and creatine phosphokinase (CK) activity, when the expression is normalized with the NF calculated with RPLPO and TBP.

## Background

The real-time polymerase chain reaction (qRT-PCR) has revolutionized the field of gene expression analysis in living organisms. In comparison to classical semi-quantitative reverse transcription-PCR (sqRT-PCR), the main advantages of qRT-PCR are its higher sensitivity, specificity, and broad quantification range [1,2]. Despite being an extremely powerful technique, qRT-PCR suffers from certain pitfalls, the most important being the normalization with a reliable reference gene, habitually called 'housekeeping gene (HKG)'. The housekeeping term was initially given to genes that are necessary for cell function and being constitutively expressed in each cell type [3] but obviously it would be more precise to call it 'reference gene (RG)'. RG are often taken from the literature and used across a variety of experimental conditions, some of which may induce differences in the RG own expression under certain conditions [3]. Thus, experimental results are highly dependent on the RG gene chosen [4]. If unrecognized, unexpected changes in RG expression could result in erroneous conclusions about real biological effects such as responses to drugs [4-6]. Such errors could be introduced at a number of stages throughout the experimental protocol (input sample, RNA extraction, reverse transcription, etc.) [1,2,7]. Therefore, it is critical to correct any errors between samples when measuring RNA expression. During the selection of an adequate RG, several indications should be taken into consideration: 1) the expression of the RG must remain constant throughout the intervention; 2) the amplification efficiency of the RG should be similar to that of the genes of interest (GOI); and 3) the abundance of the RG should be similar to that of the genes of interest [8]. Therefore, appropriate RG validation of internal references is crucial in order to avoid misinterpretations of study findings [9]. If the selected RG fluctuate randomly among samples, then subtle differences between GOI will be lost. For example, variation in presumably stable RG was shown in studies examining their expression in serum-stimulated fibroblasts [9,10].

Skeletal muscle tissue engineering aims at the reconstruction of skeletal muscle loss. The field of skeletal muscle tissue engineering has involved to create efficient muscle tissue by using the regenerative potential of stem cells and their potential for proliferation and maturation [11]. The preferred sources of cells for skeletal muscle tissue engineering applications are primary satellite cells [12]. Human satellite cells can be successfully extracted and expanded in vitro and differentiated into myofibers using different stimuli. In this context, previous reports have used conventional RG [8], but there is no data available about the validity of these RG during serum-dependent differentiation of human myoblasts in vitro. Our main GOI were differentiation markers that have unknown RNA expression under experimental conditions. Consequently, we could anticipate that differences between the treatments might be small. Therefore, it was highly important to find an internal reference with minimal variability. Thus, we used qRT-PCR to investigate the levels of six RG expressed in cultured myoblasts. Here we report the most suitable RG to detect small variations during the differentiation of myoblasts under certain culture conditions.

## Results

### Differentiation of Myoblasts

Samples indicated various stages of differentiation. We initially verified that our samples truly represented differentiating cells by analyzing the creatine phosphokinase (CK) activity level, a well-known and accepted marker of differentiation [11].

### RNA Quality

All RNA samples were examined as to their concentration, purity, and integrity. RNA purity was measured using the NanoDrop^® ^Spectrophotometer. Based on the absorbance ratio at 260 nm/280 nm (mean ± standard deviation [SD], 1.90 ± 0.05), all RNA samples were pure and protein free. The mean (± SEM) A260/230 ratio of 1.90 ± 0.20 (range from 1.65 – 2.10) indicated that the RNA was free of phenol and ethanol. RNA integrity was assessed by the calculation of RIN values using the Agilent 2100 Bioanalyzer. Differentiated and undifferentiated myoblast samples revealed RIN values between 6.0 and 8.0.

### Stability of RG within Sample Groups

First, the efficiencies of every run were calculated using standard curves. The mean of the efficiencies of genes of interest (GOI) and RG are similar in a range between 1.94 and 2.00 (Table 1).

**Table 1:** Amplification efficiencies.

**RG**	**GAPDH**	**TBP**	**RPLPO**
**Mean**	1.97	1.94	1.94
**SEM**	0.0856	0.0364	0.0336
**n**	4	3	4

**RG**	**B2M**	**ACTB**	**PPIA**

**Mean**	1.95	1.94	1.94
**SEM**	0.0426	0.0290	0.0363
**n**	4	3	3

**GOI**	**ACTA1**	**MYOG**	**MYH3**

**Mean**	1.98	1.99	2.00
**SEM**	0.0141	0.0677	0.0504
**n**	3	4	3

Mean of the amplification efficiencies of six reference genes and three genes of interest (GOI). Efficiency calculation was performed from the slopes of the calibration curve according to the equation E = 10 [-1/slope]. Abbreviations: RG, reference genes; GOI, genes of interest; SEM, arithmetic, standard error of the mean; n, number of PCR runs.

The stability of the six RG was assessed for each sample group. Gene expression levels were measured by qRT-PCR and the expression stabilities were evaluated by the three most commonly used software-based methods: 1- geNorm [13], 2- NormFinder [14] and 3-BestKeeper [15].

#### 1-GeNorm

**Figure 1:**
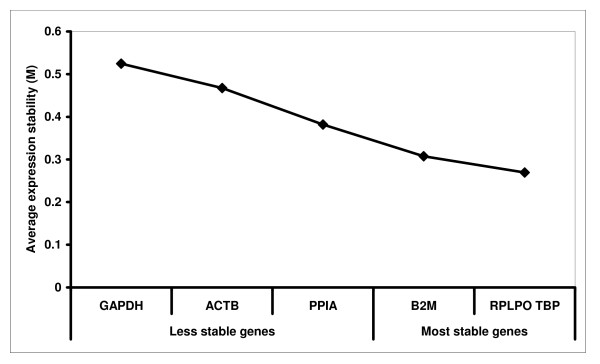
**Average expression stability values of remaining control genes**. Gene expression stability of candidate reference genes (RG) is shown in order to select the most suitable reference genes for normalization in myoblast cell cultures using geNorm analysis to identify the best RG. We performed geNorm analysis of the six selected RG, and the results are presented according to the output file of the program [13]. The least stable genes are well-established by calculating the average expression stability measure M. The value of M was calculated for each gene. The x axis from left to right indicates the ranking of the genes according to their expression stability.

Figure 1 shows the average expression stability (M). M values indicate the stability of a given gene's expression. Based on the M value, the majority of RG are below the GeNorm's arbitrary cut-off level of 0.5 for stability (Figure 1) automatically selected by the software, suggests that the use of any of these RG for normalization is applicable. Successive elimination of less stable genes according to lowest M values generated a ranking of genes and resulted in the identification of the RPLPO and TBP as the two most stable genes. In addition to the gene stability measure M, the geNorm program calculates a normalization factor (NF). The NF is determined from RPLPO and TBP genes, taking into account the variable as the pair wise variation between two sequential normalization factors (data not shown).

#### 2- NormFinder

Table 2 shows the ranking order of the six candidate reference genes mentioned above, using the NormFinder program to calculate their expression stability. Again, RPLPO and TBP were found to be the most stable genes. The NormFinder selected RPLPO as the most stable reference gene with a stability value of 0.093 (pooled data from myoblasts cultured in normal [N] or differentiation [D] medium) and 0.071 when only data of myoblasts cultured in medium N were analyzed (Table 1). RPLPO and TBP were identified as the best genes from myoblasts cultured in medium D with a stability value of 0.050 (Table 2).

**Table 2:** Expression Stability of RG Evaluated by NormFinder Software

**Gene name**	**N and D**		**Medium N**		**Medium D**	
	
	Stability value*	**SE**	Stability value*	**SE**	Stability value*	**SE**
**RPLPO**	0.093	0.069	0.071	0.079	0.050	0.135

**TBP**	0.109	0.065	0.100	0.072	0.050	0.135

**PPIA**	0.318	0.085	0.201	0.087	0.335	0.131

**GAPDH**	0.374	0.096	0.376	0.138	0.393	0.148

**B2M**	0.258	0.075	0.181	0.083	0.125	0.089

**ACTB**	0.344	0.090	0.205	0.088	0.348	0.135

**Best gene:**	RPLPO		RPLPO		RPLPO and TBP	

Candidate reference genes for normalization are listed according to their expression stability calculated by the NormFinder program (14). The analysis of the expression stability was performed taken the expression of the RG from RNA samples of myoblasts. The analysis was performed using myoblasts cultured in normal medium (N), or differentiation medium (D). Additionally, the analysis of the combination of N and D together is shown. *High expression stability is indicated by a low stability value as an estimate of the combined intra- and intergroup variation of the individual gene. SE stands for standard error. Representative results from three experiments.

#### 3- BestKeeper (BK)

The results of reference gene evaluation by the BestKeeper tool are shown in Table 3. According to the variability observed, RG can be identified as the most stable genes, as they exhibited the lowest coefficient of variance (CV ± SD). In this context, we found RPLPO, TBP or PPIA, with CV ± SD of 4.5 ± 0.83, 4.0 ± 0.99 or 2.9 ± 0.7, respectively, to be the most stable reference genes (Table 3). B2M, ACTB and GAPDH exhibited the highest coefficient of variance with 5.8 ± 1.05, 6.0 ± 1.16 or 7.5 ± 1.12, indicating that these were the least stable NormFinder RG (Table 3, bold italic letters). However, genes that show a SD higher than 1 (= starting template variation by the factor 2) should be considered unacceptable [15]. A low SD of the crossing point (CP) values should be expected for useful RG. Corresponding to the estimation of the SD (± CP) of the CV [% CP], value was highest for ACTB, GAPDH and B2M (Table 3, bold italic letters). This constitutes a reason to exclude these genes from the BestKeeper index calculation, as they are not reliable RG in this setting [15]. The weighted index BestKeeper calculated for the six candidates showed a SD of CP = ± 0.98 cycles. After the exclusion of GAPDH, B2M, and ACTB from the index, its variation decreased (SD = ± 0.83 cycles) to a figure identical to RPLPO (Table 3). GOI expression data are statistically processed with the BestKeeper software in the same way as those of RG, e.g., their GM, AM, SD, CV, Min. and Max. values (Table 4). According to the variability observed, GOI can be identified as well as RG by choosing the most stable genes with the lowest variation, but none of the GOI tested (ACTA1, MYOG and MYH3) showed an acceptable CV ± SD variability (13.77 ± 3.06, 11.62 ± 2.88 and 9.11 ± 2.75 respectively, bold italic letters). A new version of the BestKeeper tool that also employs non-parametric methods is being prepared [15] by which genes with very different expression levels can be compared. On the other hand, all six RG correlated very well one with another (results not shown).

**Table 3:** Expression Stability of RG Evaluated by BestKeeper Software

	**PPIA**	**RPLPO**	**TBP**	**B2M**	**GAPDH**	**ACTB**	**BK** **n = 6**	**BK** **n = 3**
**n**	10.0	10.0	10.0	10.0	10.0	10.0	10.0	10.0

**SD [± CP]**	0.70	0.83	0.99	***1.05***	***1.16***	***1.12***	0.98	0.83

**CV [% CP]**	2.9	4.5	4.0	***5.8***	***6.0***	***7.5***	5.0	3.7

Descriptive statistics of six RG based on their crossing point (CP) values. In the two last columns the BestKeeper (BK) index is computed together with the same descriptive parameters, either for six genes (RPLPO, TBP, GAPDH, B2M, ACTB and PPIA) or for three genes after removal of GAPDH, B2M and ACTB. Abbreviations: CV [%CP]: the coefficient of variance expressed as a percentage on the CP level; SD [± CP]: the standard deviation of the CP; SD [± x-fold]: standard deviation of the absolute regulation coefficients; Results from three independent experiments are shown.

**Table 4:** Expression Stability of GOI Evaluated by BestKeeper Software

	**ACTA1**	**MYOG**	**MYH3**
**n**	10.00	10.00	10.00

**GM [CP]**	21.95	24.56	30.07

**AM [CP]**	22.21	24.78	30.21

**min [CP]**	16.92	20.10	27.05

**max [CP]**	27.14	30.06	35.25

**SD [± CP]**	***3.06***	***2.88***	***2.75***

**CV [% CP]**	***13.77***	***11.62***	***9.11***

**min [x-fold]**	-33.99	-21.40	-8.49

**max [x-fold]**	38.16	43.48	39.50

**SD [± x-fold]**	8.55	7.21	7.04

Descriptive statistics of three GOI based on their crossing point (CP) values. Abbreviations: AM [CP]: the arithmetic mean of CP; CV [%CP]: the coefficient of variance expressed as a percentage on the CP level; GM [CP]: the geometric mean of CP; min [CP] and max [CP]: the extreme values of CP; Min [x-fold] and Max [x-fold]: the extreme values of expression levels expressed as an absolute x-fold over- or under-regulation coefficient; n: number of samples; SD [± CP]: the standard deviation of the CP; SD [± x-fold]: standard deviation of the absolute regulation coefficients. Results from three independent experiments are shown.

### Evaluation of Selected Candidate RG and Normalization Approach

Following identification of the most stable RG and the normalization factor (NF) from the full panel of RG, a method was needed for their evaluation. Correlations among the status of the myoblast differentiation (identified by CK activity) and expression of RG and ACTA1, MYOG and MYH3 (normalized with NF [RPLPO+TBP]) are shown in Table 5. Therefore, we expected these observations to be mirrored at the mRNA level. More specifically, following treatment with the differentiation medium, a high and significant correlation between the CK activity and the expression of ACTA1, MYOG or MYH3 was found (Table 5), whereas RPLPO, TBP and GAPDH showed no significant correlation with CK (Table 5, bold italic letters). However, both positive (B2M) and negative (ACTB) correlation between gene expression and CK were also found (Table 5). A significant positive correlation was found between the GOI (Table 5), whereas there was no correlation between RPLPO, TBP or GAPDH and ACTA1, MYOG or MYH3. However, B2M showed a good correlation (Table 5). ACTB has shown a negative correlation with ACTA1 and MYH3 (Table 5) but not with MYOG (Table 5, bold italic letters).

**Table 5:** Correlation Between CK Activity, RG and Differentiation Markers

		**CK**	**ACTA1/NF**	**MYOG/NF**	**MYH3/NF**
**RPLPO/NF**	**r**	***-0.144***	***-0.435***	***-0.522***	***-0.164***
	**p<**	***0.692***	***0.208***	***0.122***	***0.65***
	**n**	***10***	***10***	***10***	***10***

**TBP/NF**	**r**	***0.138***	***0.453***	***0.547***	***0.143***
	**p<**	***0.704***	***0.188***	***0.102***	***0.694***
	**n**	***10***	***10***	***10***	***10***

**GAPDH/NF**	**r**	***-0.148***	***-0.405***	***-0.312***	***-0.306***
	**p<**	***0.683***	***0.245***	***0.379***	***0.39***
	**n**	***10***	***10***	***10***	***10***

**B2M/NF**	**r**	0.85	0.83	0.788	0.9
	**p<**	0.00183	0.00293	0.00674	0.000384
	**n**	10	10	10	10

**ACTB/NF**	**r**	-0.757	-0.629	-0.514	-0.768
	**p<**	0.0112	0.0514	0.129	0.00952
	**n**	10	10	10	10

**PPIA/NF**	**r**	***-0.127***	***-0.291***	***-0.41***	***0.0294***
	**p<**	***0.726***	***0.414***	***0.239***	***0.936***
	**n**	***10***	***10***	***10***	***10***

**MYH3/NF**	**r**	0.824	0.754	0.677	
	**p<**	0.00341	0.0117	0.0314	
	**n**	10	10	10	

**MYOG/NF**	**r**	0.743	0.956		
	**p<**	0.0139	0.000015		
	**n**	10	10		

**ACTA1/NF**	**r**	0.766			
	**p<**	0.00974			
	**n**	10			

Pearson's correlation between the state of the myoblast differentiation (CK activity) and expression of the ACTA1, MYOG or MYH3 normalized with NF calculated with RPLPO and TBP by GeNorm and the RG normalized with the same NF. Abbreviations: CK: protein kinase activity; n: number of samples; p: significance; r: correlation coefficient. Bold italic letters indicate correlation which is not statistically significant. Results from three independent experiments are shown.

**Figure 2:**
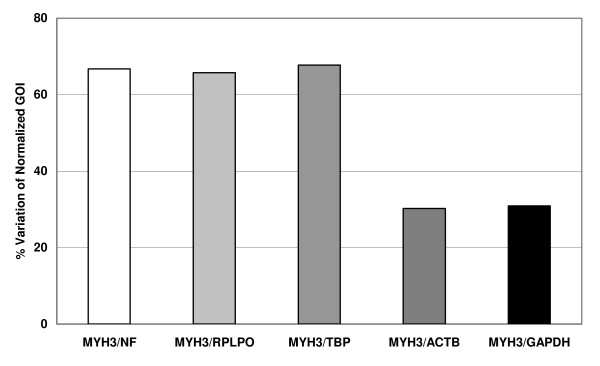
**Percent variation of gene of interest (GOI) expression**. Percent variation in the expression of MYH3 gene between myoblasts treated with medium N and medium D after 12 days culture. The MYH3 expression was normalized with the NF, the best RG (RPLPO and TBP) and the worst RG (ACTB and GAPDH) candidates. Representative results from at least three experiments.

Figure 2 shows the percent of GOI variation normalized with NF and the best and worst RG candidates, after 12 days myoblasts differentiation. When the GOI were normalized with NF, RPLPO and TBP, we identify approximately 67.0% of variation; in contrast only 30.0% variation was observed, when MYH3 was normalized with ACTB or GAPDH.

## Discussion

Myogenesis of skeletal muscle is a highly complex phenomenon controlled and regulated by multiple intracellular signalling mechanisms [16]. In order to investigate optimal culture conditions and characterization of human myoblasts, we analyzed cell behaviour under different culture media and showed that cultures treated with differentiation medium expressed several characteristic features of mature skeletal muscle [11]. Furthermore, in myoblasts cultured with growth medium, certain markers of differentiation were detected, demonstrating that, for an accurate characterization, multiple markers (e.g. genes, enzymes and structure proteins) must be analyzed [11]. During skeletal muscle differentiation, mononucleated proliferating myoblasts stop dividing, activate muscle-specific gene expression, and fuse into multinucleated myotubes [11]; these events can produce drastic or small variation in the expression of genes and proteins. Markers of differentiation were also detected in cultures treated with non-differentiation medium, but there was no formation of myotubes. In the enzymatic assay of CK, a well-known and accepted marker of differentiation, cultures treated with differentiation medium showed a higher activity, evidencing a higher degree of differentiation [11]. In this context, the strategy used to normalize the mRNA with adequate RG is critical. To identify the best RG as normalisers in gene expression studies, several strategies including computer programs have been recommended [5,6,9,14-20]. The absence of the differential expression or variability of the candidate RG examined is the strongest proof of suitability and stability [20]. Therefore, we proposed to use several software programs to analyze six RG and select the RG or normalization factors to correct the RNA input. The major result of our study – evaluating six candidate RG in myoblasts under normal and differentiation conditions, during 8 and 12 days of treatment – was that the genes RPLPO or TBP were identified by the three independent software programs as suitable RG that did not differ in their expression in differentiated and undifferentiated myoblasts. Consequently, only RPLPO, identified by geNorm, NormFinder and BestKeeper software programs fulfils the criterion of expression stability between samples and can be recommended as an accurate normalizer for relative gene quantification in cultured myoblasts. First, the expression of the candidate RG between the respective conditions was compared using an adequate test such as geNorm, NormFinder and BestKeeper. It can be assumed that genes with significantly different expressions are not suited for target gene normalization, since they are affected by the study condition of interest. Thus, those genes should be excluded as normalizers. In the present study, the expression of all genes in the differentiated and undifferentiated myoblast samples showed different grades of variability but the most stable were RPLPO and TBP. We used the BestKeeper software to confirm the results obtained by geNorm and NormFinder. The raw CPs analyzed by BestKeeper seem to be good estimators of the expression levels as they are (in most cases) normally distributed and a parametric test can thus be performed [15]. This also gives the CP datasets the Gaussian distribution, justifying usage of parametric methods [15]. Low numbers of expressed genes where CPs were obtained somewhere surely show different variances compared to highly expressed genes with CPs. These higher levels of CV and SD invalidate the use of BestKeeper as a reference to normalizing the expression of GOI [15]. The correlation analysis of the normalized gene with NF showed a bad correlation between the RG RPLPO, TBP or GAPDH and the different differentiation markers (CK, ACTA1, MYOG and MYH3). In this context, the absence of correlation confirmed that the RG selected by geNorm, NormFinder and BestKeeper are suitable as reference for the normalization. In addition, we found a significant correlation between B2M or ACTB and the GOI which indicates a variation associated with the differentiation status of the myoblast. Thus, they cannot be used as reference genes.

Two or three genes represent a realistic calculation basis in a common laboratory and the minimum necessary number for a good analysis [15]. RPLPO STD dev CP [± SD] is identical to BestKeeper calculated with RPLPO, TBP and PPIA. In this context, the RG, RPLPO and TBP, detected by BestKeeper agree with the best RG detected by geNorm and NormFinder.

The normalization with NF, RPLPO or TBP allows measurements of differences in the expression of the differentiation gene-markers between myoblasts treated with differentiation medium or normal medium (control).

## Conclusion

In conclusion, the RG RPLPO, TBP as well as a NF calculated by geNorm software are recommended as references for relative gene quantification in gene profiling studies of single genes in myoblasts under serum-differentiation conditions. The confirmation of RG by the three software programs is a suitable method to perform an adequate normalization of the mRNA input in quantitative PCR experiments.

## Methods

### Cell Culture

Human skeletal muscle biopsies were obtained from 15 patients during head and neck surgery. The median age of these patients was 58, ranging from 41 to 72. The study was approved by the Ethics Committee of the Mannheim Faculty of Medicine, University of Heidelberg, Germany, and the patients gave informed consent. Satellite cells were dissociated from the minced muscles by digestion with collagenase B (Roche, Mannheim, Germany) for 60 min and 0.05% trypsin-0.02% EDTA (Promo Cell, Heidelberg, Germany) for 45 min at 37°C, filtered through a sterile 70-μm cell strainer (Becton Dickinson, Franklin Lakes, NJ, USA) and purified with the pre-plating technique as recently described [12]. Purity of myoblast cultures (>80%) was evaluated by anti-desmin immunostainings. Cells were grown on 0.2% gelatine-coated culture flasks (Sigma, Deisenhofen, Germany) and in Ham's F10 growth medium, containing 1% penicillin/streptomycin/fungizone-solution (PSF), 2 mM L-glutamine (all from PromoCell, Heidelberg, Germany) and 10% foetal bovine serum (FBS) (PAA Laboratories, Linz, Austria) (GM). Cells were maintained at 37°C in a humidified atmosphere of 5% CO2 and 95% air, and the medium was changed every 72 h.

### Differentiation of skeletal muscle myoblasts

Skeletal myoblasts were cultured to ~60% confluence, washed with phosphate-buffered saline (PBS) and induced to differentiate by a change in medium (D) consisting of minimal essential medium (MEM) (PromoCell) supplemented with 2% horse serum (PAA Laboratories), 2 mM L-glutamine and PSF (both from PromoCell).

### RNA Isolation

Total RNA was isolated with the RNA Mini Kit (Qiagen, Hilden, Germany), according to the manufacturer's instructions. RNA concentration, purity, and integrity was determined by A260 and A280 (A260/A280 = 1.7–2.0) measurements using a NanoDrop 8000 Spectrophotometer (Thermo Scientific, Schwerte, Germany) and Agilent 2100 Bioanalyzer (Agilent Technologies, Waldbronn, Germany).

### cDNA Synthesis and Real-time PCR

An aliquot of 0.5 μg total RNA was treated with 1 unit DNAse (Fermentas, St. Leon-Rot, Germany) 30 min at 37°C. Reverse transcription of RNA (0.5 μg) was performed with oligo (dT)12–18 primer and 200 units of SUPERSCRIPT II (Invitrogen, Karlsruhe, Germany) and 24 units of Ribo LockTM RNAse inhibitor (Fermentas) for 1 h at 42°C. The cDNA was used for PCR analysis. All cDNA probes were analyzed for: ACTA1, (NM_001100), amplicon length 85 bp; MYOG, (NM_002479), amplicon length 113 bp; MYH3, (NM_002470), amplicon length 84 bp and the RG: ACTB (NM_001101), amplicon length 104 bp; B2M, (NM_004048), amplicon length 98 bp; GAPDH, (NM_002046), amplicon length 119 bp; cyclophilin A/PPIA, (NM_203430), amplicon length 121 bp; RPLPO, (NM_001002), amplicon length 170 bp; TBP, (NM_003194), amplicon length 132 bp. The QuantiTect/PrimerAssays were purchased from QIAGEN GmbH (Hilden, Germany). cDNAs were amplified with Brilliant^® ^II SYBR^® ^Green QRT-PCR Master Mix (Stratagene-Agilent Technologies, Waldbronn, Germany). The thermal profile consisted of 1 cycle at 50°C for 2 minutes followed by 1 cycle at 95°C (2 min), 45 cycles at 95°C (15 sec), 60°C (1 min). Amplification was performed using the Mx3005P™ QPCR System (Stratagene). For relative quantification, a standard curve was generated in every individual run. Shortly, total RNA was pooled from muscle biopsies of healthy human volunteers, reverse transcription was performed and a serial dilution of the cDNA was used to perform the calibration curve. The data were analyzed using the relative standard curve method. For each unknown sample, the relative amount is calculated using linear regression analysis from their respective standard curves. Data were analyzed using the Mx3005P analysis software (Stratagene-Agilent Technologies, Waldbronn, Germany). The efficiencies of all GOI and RG were calculated in every individual run (Table 1).

### Creatine Phosphokinase (CK) Analysis

Differentiation of myoblasts was identified by measuring the creatine phosphokinase (CK) activity of developing myofibers using the CK assay (Sigma-Aldrich Chemie, Taufkirchen, Germany). Cells were lysed with a buffer containing NP-40, protease inhibitors and were stored at -80°C. Samples were assayed according to the manufacturer's protocol. Enzymatic activity was normalized over the total protein content determined by the Lowry protein assay (Bio-Rad, Munich, Germany).

### Analysis of Expression Stability

For stability comparisons of candidate RG within sample groups, the software geNorm, version 3.4 [13] (Visual Basic application tool for Microsoft Excel), NormFinder [14] and BestKeeper [15], were used according to developer's recommendations.

GeNorm uses a gene-stability measure M, which is defined as the average pair wise variation between a particular gene and all other control genes. It calculates the optimal number of genes necessary for normalization of a target gene and combines them into a normalization factor (NF). The NormFinder program uses a model-based approach for estimation of expression and enables the identification of the single best genes as well as giving a ranking order. The BestKeeper software determines the best suited reference genes and combines them into an index (BK). The index can be compared with further target genes to decide whether they are differentially expressed under certain conditions. In addition, the option in NormFinder to define groups was applied to compare the effect of the treatment of myoblasts with differentiation medium.

### Statistical analysis

The software Sigma Plot was used to carry out statistical analysis, by paired Student's t-test or Mann-Whitney-U test, as well as Pearson's product moment correlation test of gene expression among experimental groups. Gene expression results were expressed as the mean. P < 0.05 was considered statistically significant.

## Authors' contributions

JSS carried out the cell biologic studies and drafted the manuscript. GAB carried out the molecular biologic studies and drafted the manuscript. KH participated in the coordination of the study. RK participated in the design and coordination of the study and drafted the manuscript. URG conceived and designed the study and drafted the manuscript. All authors read and approved the final manuscript.
